# Supplementary material for: Association between oral microbiome and seven types of cancers in East Asian population: a two-sample Mendelian randomization analysis
Source: Front Mol Biosci. 2023 Nov 21;10:1327893. doi: 10.3389/fmolb.2023.1327893 (PMC10702768; doi:10.3389/fmolb.2023.1327893)
Supplement: Supplementary file 1 [file DataSheet1.ZIP › Supplementary Materials/MR plots for tongue/tongue═╝/Pancreatic cancer/╥╚╧┘░⌐_╔╕╤í╜ß╣√/pheno.2687_to_pancreatic cancer_forest.pdf]

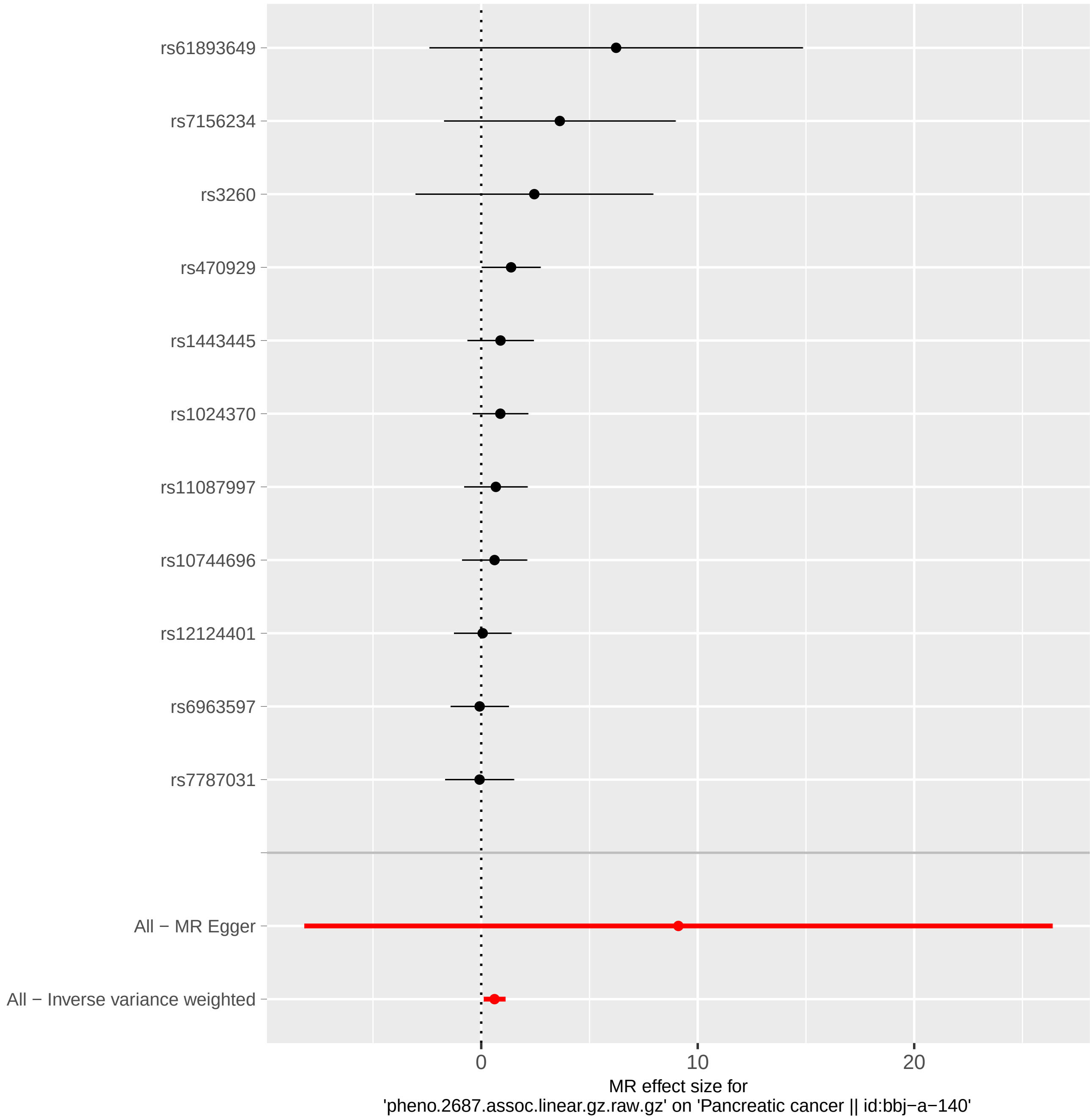

rs61893649
rs7156234
rs3260
rs470929
rs1443445
rs1024370
rs11087997
rs10744696
rs12124401
rs6963597
rs7787031
All − MR Egger
All − Inverse variance weighted
0
10
20
MR effect size for
'pheno.2687.assoc.linear.gz.raw.gz' on 'Pancreatic cancer || id:bbj−a−140'
